# Numerical Study on Influence of Cross Flow on Rewetting of AHWR Fuel Bundle

**DOI:** 10.1155/2014/589543

**Published:** 2014-02-02

**Authors:** Mithilesh Kumar, D. Mukhopadhyay, A. K. Ghosh, Ravi Kumar

**Affiliations:** ^1^Reactor Safety Division, Bhabha Atomic Research Centre, Mumbai, India; ^2^Raja Ramanna Fellow, Bhabha Atomic Research Centre, Mumbai, India; ^3^Indian Institute of Technology, Roorkee, India

## Abstract

Numerical study on AHWR fuel bundle has been carried out to assess influence of circumferential and cross flow rewetting on the conduction heat transfer. The AHWR fuel bundle quenching under accident condition is designed primarily with radial jets at several axial locations. A 3D (*r*, *θ*, *z*) transient conduction fuel pin model has been developed to carry out the study with a finite difference method (FDM) technique with alternating direction implicit (ADI) scheme. The single pin has been considered to study effect of circumferential conduction and multipins have been considered to study the influence of cross flow. Both analyses are carried out with the same fluid temperature and heat transfer coefficients as boundary conditions. It has been found from the analyses that, for radial jet, the circumferential conduction is significant and due to influence of overall cross flow the reductions in fuel temperature in the same quench plane in different rings are different with same initial surface temperature. Influence of cross flow on rewetting is found to be very significant. Outer fuel pins rewetting time is higher than inner.

## 1. Introduction

Rewetting of hot surface is a process in which a liquid wets a hot surface by displacing its own vapour that otherwise prevents the contact between the solid and liquid phases. This has generated immense interest in studying rewetting through both theoretical simulation by Yamanouchi [1] and Coney [2] and experimental study which was carriedout by Yamanouchi [1] and Duffey and Porthouse [3]. Falling film rewetting for several vertical geometries such as plates [2, 4], rods by Blair [5], and tubes by Satapathy and Sahoo [6] had been modeled by a number of researchers. In general, in all models, a moving rewetting front that divides the solid into two distinct regions is considered. Most of the models also consider a constant rewetting velocity that reduces the problem into a quasistatic one. Initial efforts were made to formulate one-dimensional conduction models by Yamanouchi [1] that are reasonably successful in correlating rewetting phenomena at low Peclet number. Tien and Yao [4] presented the asymptotic solutions of a two-dimensional conduction model which clearly demonstrates the different physical pictures for the cases of high and low coolant flow rates. A variety of techniques have been used for solving two-dimensional conduction models for falling film rewetting. Some of the important studies are elaborated. Because of mathematical difficulty, most two-dimensional analyses are either approximate or numerical ones. Duffey and Porthouse [3] first considered solving the rewetting problem by separation of variables. They retained only the first term in the series solution. However, Coney [2] reported that using a small number of terms in a series yields inaccurate results due to slow convergence of the series. An approximate solution to the same model for a cylindrical rod was presented by Blair [5]. Tien and Yao [4] first applied the Wiener-Hopf technique to a two-dimensional rewetting problem of a rectangular slab, while an exact solution to the same problem was presented by Castiglia et al. [7], employing the method of separation of variables. Numerical solutions of conduction controlled rewetting were provided by Satapathy and Sahoo [6], Thompson [8], and Raj and Date [9] by using the finite difference technique. Heat balance integral method (HBIM) is one of many semianalytical methods used to solve conduction problems by Eckert et al. [10, 11]. This is analogous to classical integral technique used for fluid flow and convective heat transfer analysis. A numerical study has been made to investigate the effect of internal heating and precursory cooling during quenching of an infinite tube and was studied by Satapathy and Sahoo [6]. An experimental assessment of rewetting of an advanced heavy water reactor (AHWR) specific nuclear fuel bundle with radial jets has been carried out by Patil et al. [12]. The experimentation involved cooling of a 54-rod bundle by in-bundle injection and demonstrated that quenching occurs for all fuel pins along the radial direction. But maximum temperature is limited to be 400°C and no heat generation has been considered during the quenching of experiment.

**Figure 1:**
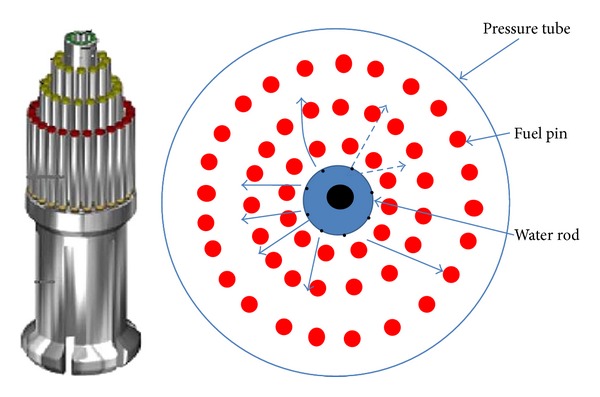
View of AHWR fuel bundle.

The numerical study presented in this paper aims to bring out a comparison in rewetting pattern for a radial jet rewetting versus axial rewetting for AHWR fuel bundle. The proposed reactor is a vertical pressure tube type, heavy water moderated boiling light water coolant reactor. Fuel bundle is housed in the pressure tube (PT) which in turn is housed in calandria tube (CT). The 3.5 m height fuel bundle is having 54 fuel pins arranged in three different concentric rings as shown in Figure 1. The inner, middle, and outer rings have 12, 18, and 24 fuel pins and centre of the fuel pin has a water rod which is used to inject emergency coolant in radial direction at different elevations of the fuel bundle under a pipe break scenario. The water rod is having eight holes of 1.5 mm dia at one elevation along the circumference of water rod. The radial injection is designed for 13 different axial locations along the 3.5 m length of water rod. More details of AHWR fuel bundle and its injection arrangement are furnished by Sinha and Kakodkar [13]. Bottom reflooding is also an option with the designer where water may be injected from bottom most holes instead on 13 axial elevations.

Under this study, a 3D (*r*, *θ*, *z*) transient conduction fuel pin model has been developed based on finite difference method (FDM) technique with alternating direction implicit (ADI) scheme. The number of nodes in *r*, *θ*, and *z*-direction has been considered to be 50, 20, and 50, respectively. A 1.6 ms time step has been used as predicted from stability criteria. A total time of 4 hrs is required for individual rewetting study.

Single pin from first circle has been considered for which bottom and radial reflooding are studied. The 1st circle pin is selected as it experiences a strong radial jet and experiences a large circumferential temperature gradient. For bottom rewetting case all the pins will experience the same velocity front irrespective of fuel pin location. However, the developed computational model is equally applicable for 2nd and 3rd circle pins with different boundary conditions. Both analyses are carried out with same fluid temperature and heat transfer coefficients as boundary conditions. The single fuel pin has been considered for circumferential and axial rewetting. The three-dimensional partial differential equation for unsteady state conduction equation for cylindrical rod is as follows:

(1) $$\begin{array}{c} \frac{\partial^{2}T}{\partial r^{2}}+\frac{1}{r}\frac{\partial T}{\partial r}+\frac{1}{r^{2}}\frac{\partial^{2}T}{\partial \theta^{2}}+\frac{\partial^{2}T}{\partial z^{2}}+\frac{Q^{\prime \prime \prime }}{K}=\frac{1}{\alpha }\frac{\partial T}{\partial t}. \end{array}$$

**Figure 2:**
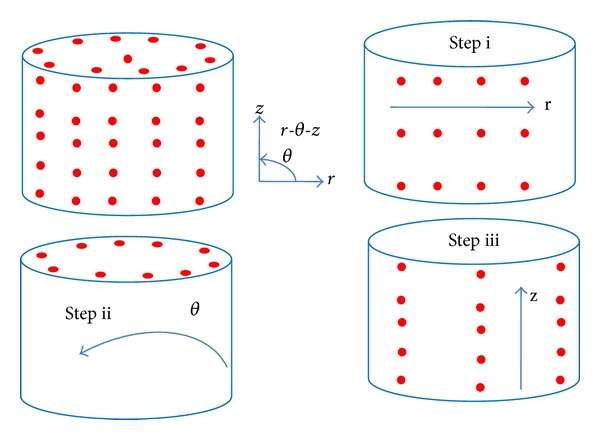
ADI steps to solve the conduction equation.

Alternating direction implicit (ADI) numerical method has been adopted. With an ADI method the heat diffusion equation is first solved implicitly in the *r*-direction while leaving the other two directions explicit. The heat diffusion equation is then solved implicitly in a similar way in the *θ*- and *z*-direction. This scheme reduces a three-dimensional problem to a series of one-dimensional implicit problems.  Figure 2 shows the three different steps in ADI.

Many methods [14–17] are also available in ADI for solving the heat diffusion equation, such as Peaceman-Rachford pure-ADI method, Brain method, Douglas method, and the method based on superposition principle by Jules Thibault [18]. All above schemes having the same problem of conditionally stable criteria as right side of equations have a negative coefficient. This paper discusses conduction effect on axial, circumferential direction as well as influence of rewetting of fuel pins in different concentric circles due to cross flow model.

The tridiagonal matrix has been used to solve the equation which corresponds to given direction. Normally in ADI scheme, first solution is obtained in *r*-direction in an implicit manner and leaves both directions explicit. But here all the possible six combinations, like *r*-*θ*-*z*, *r*-*z*-*θ*, *θ*-*r*-*z*, *θ*-*z*-*r*, *z*-*r*-*θ*, and *z*-*θ*-*r*, have been used to solve the heat diffusion equation and compared results.

## 2. 3D Model Development

### 2.1. Finite Difference Formulation

FDM is used to formulate the heat diffusion equation in cylindrical coordinate (see (1)) and further semi-implicit method used to formulate in *r*-*θ*-*z*-direction. The differential equation has been divided into the three categories (1) centre of fuel pin: (2) between centre of fuel pin and surface, and (3) surface of fuel pin. The length increment in *r*, *θ*, *z*-directions are Δ*r*, Δ*θ*, Δ*z*, respectively. The semi-implicit scheme has been used to formulate the differential equation in *r*, *θ*, and *z*-directions and is represented by (2), (5), and (8) respectfully. As per ADI norms, first 1/3 time increment has been taken to solve the temperature at different grid points in *r*-direction, next 1/3 time increment for solving the temperature in *θ*-direction, and last time step for solving the temperature in *z*-direction. For stability criteria, the coefficient of *T*_*i*,*j*,*k*_^*n*+1/3^, *T*_*i*,*j*,*k*_^*n*+2/3^, *T*_*i*,*j*,*k*_^*n*+1^ in right side of the equation must be positive.

Finite difference formulation between centre and surface of fuel rod: *r*-direction

(2) Ti+1,j,kn+1/3−2Ti,j,kn+1/3+Ti−1,j,kn+1/3Δr2+1riTi+1,j,kn+1/3−Ti−1,j,kn+1/32Δr +Ti,j+1,kn−2Ti,j,kn+Ti,j−1,knri2Δθ2+Ti,j,k+1n−2Ti,j,kn+Ti,j,k−1nΔz2 +Q′′′k=1αfTi,j,kn+1/3−Ti,j,knΔt/3,

or

(3) (1+2F0Δt)Ti,j,kn+1/3=F0Δt(1−Δr2ri)Ti−1,j,kn+1/3+F0Δt(1+Δr2ri)Ti+1,j,kn+1/3+(1−2FθΔtri2−2FzΔt)Ti,j,kn+FθΔtri2(Ti,j+1,kn+Ti,j−1,kn)+FzΔt(Ti,j,k+1n+Ti,j,k−1n)+Q′′′Δt3ρfCp.

Condition for stability

(4) $$\begin{array}{c} \left(1-\frac{2F_{\theta }\Delta t}{r_{i}^{2}}-2F_{z}\Delta t\right)\geq 0. \end{array}$$

*θ*-direction

(5) Ti+1,j,kn+1/3−2Ti,j,kn+1/3+Ti−1,j,kn+1/3Δr2+1riTi+1,j,kn+1/3−Ti−1,j,kn+1/32Δr +Ti,j+1,kn+2/3−2Ti,j,kn+2/3+Ti,j−1,kn+2/3ri2Δθ2 +Ti,j,k+1n+1/3−2Ti,j,kn+1/3+Ti,j,k−1n+1/3Δz2 +Q′′′k=1αfTi,j,kn+2/3−Ti,j,kn+1/3Δt/3,

or

(6) (1+2FθΔtri2)Ti,j,kn+2/3 =FθΔtri2(Ti,j+1,kn+2/3+Ti,j−1,kn+2/3)  +(1−2F0Δt−2FzΔt)Ti,j,kn+1/3+F0Δt(1+Δr2ri)Ti+1,j,kn+1/3  +F0Δt(1−Δr2ri)Ti−1,j,kn+1/3  +FzΔt(Ti,j,k+1n+1/3+Ti,j,k−1n+1/3)+Q′′′Δt3ρfCp.

Condition for stability

(7) $$\begin{array}{c} \left(1-2F_{0}\Delta t-2F_{z}\Delta t\right)\geq 0. \end{array}$$

*z*-direction

(8) Ti+1,j,kn+2/3−2Ti,j,kn+2/3+Ti−1,j,kn+2/3Δr2+1riTi+1,j,kn+2/3−Ti−1,j,kn+2/32Δr +Ti,j+1,kn+2/3−2Ti,j,kn+2/3+Ti,j−1,kn+2/3ri2Δθ2+Ti,j,k+1n+1−2Ti,j,kn+1+Ti,j,k−1n+1Δz2 +Q′′′k=1αfTi,j,kn+1−Ti,j,kn+2/3Δt/3,

or

(9) Ti,j,kn+1(1+2FzΔt) =FzΔt(Ti,j,k+1n+1+Ti,j,k−1n+1)  +F0Δt(1+Δr2ri)Ti+1,j,kn+2/3+F0Δt(1−Δr2ri)Ti−1,j,kn+2/3  +(1−2F0Δt−2FθΔtri2)Ti,j,kn+2/3  +FθΔtri2(Ti,j+1,kn+2/3+Ti,j−1,kn+2/3)+Q′′′Δt3ρfCp.

Condition for stability

(10) $$\begin{array}{c} \left(1-2F_{0}\Delta t-\frac{2F_{\theta }\Delta t}{{r_{i}}^{2}}\right)\geq 0. \end{array}$$

### 2.2. Centre and Surface of the Fuel Pin

**Figure 3:**
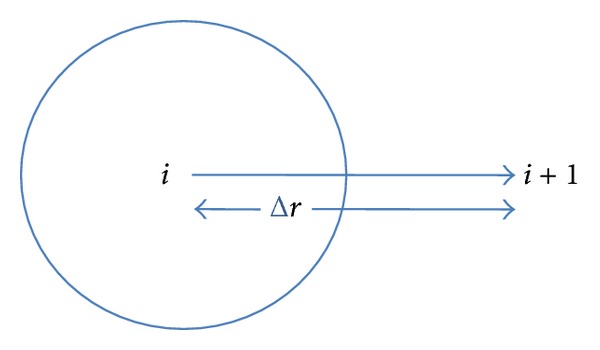
Discretisation for centre line temperature.

The centre line temperature for fuel pin has been calculated by using the concept of energy balance and finite difference formulation is used for calculating the centre line temperature. It is shown by (11). The centre line grid point is represented by *i* and next to centre line temperature is represented by *i* + 1 and is shown in Figure 3. In a similar way, surface temperature of fuel pin has been formulated in (14):

(11) Q′′′π(Δr2)2−2πks(Δr2)(Ti,j,kn−Ti+1nΔr) =π(Δr2)2ρfCp(Ti,j,kn+1−Ti,j,knΔt),

or

(12) $$\begin{array}{c} T_{i,j,k}^{n+1}=4F_{0}T_{i+1,j+1,k+1}^{n}+T_{i,j,k}^{n}\left(1-4F_{0}\right)+\frac{Q^{\prime \prime \prime }\Delta t}{\rho_{f}C_{p}}. \end{array}$$

Condition for stability

(13) $$\begin{array}{c} \left(1-4F_{0}\right)\geq 0. \end{array}$$

At surface of fuel pin.

Implicit Scheme

(14) 2πRfKf(Ti−1n+1/3−Tin+1/3)Δr+2πRfho(Tinf⁡−Tin+1/3)  +π(Rf2−(Rf−Δr2)2)Q′′′ =ρfCpπ(Rf2−(Rf−Δr2)2)(Tin+1/3−Tin)Δt/3,

or

(15) (1.0+8F0RfΔt(4Rf−Δr)+8F0RfhoΔrΔtKf(4Rf−Δr))Tin+1/3 =8F0RfΔt(4Rf−Δr)Ti−1n+1+Q′′′αΔt3Kf+8F0RfhoΔrΔtKf(4Rf−Δr)Tinf⁡+Tin.

### 2.3. Boundary Condition and Solver

Once the formulation of differential equations is over then, with help of boundary, it is required to calculate the temperature at different nodes of fuel pin. There are two cases considered for validation of numerical code; first case is constant coolant temperature and heat transfer coefficient and second case is coolant temperature varies along the length of fuel pin when it flows along the length of pin. The governing equation which calculates the coolant average temperature between the corresponding nodes of fuel pin is represented by (17).

Energy balance at surface of fuel pin

(16) $$\begin{array}{c} h_{o}A\left(T_{s}-T_{\inf}\right)=mC_{p}\left(T_{\text{out}}-T_{\text{in}}\right), \end{array}$$

(17) $$\begin{array}{c} T_{\inf}=\frac{T_{\text{in}}+T_{\text{out}}}{2}, \end{array}$$

(18) $$\begin{array}{c} T_{\text{out}}\left(\frac{h_{o}A}{2mC_{p}}+1\right)=\frac{h_{o}AT_{s}}{mC_{p}}+\left(1-\frac{h_{o}A}{2mC_{p}}\right)T_{\text{in}}. \end{array}$$

Equation (18) is used for calculating the outlet coolant temperature for a given volume. After applying the boundary condition, it is required to solve the discretised partial differential in each direction by ADI method. At each incremental time step, the differential equations form a tridiagonal matrix and are solved by Thomas algorithm (see (19)). In first time increment (i.e., 1/3 s) it solves temperature in *r*-direction at different grids points and takes old values of temperature in *θ*, *z*-direction. Similarly for higher time step (2/3 s), this algorithm solves in *θ*-direction and remaining directions take previous value:

(19) $$\begin{array}{c} \left[\begin{array}{ccccccc} \circ & \circ & & & & & \\ {}* & * & * & & & & \\ & * & * & * & & & \\ & & * & * & * & & \\ & & & & : & & \\ & & & & : & & \\ & & & & * & * & *\\ & & & & & \circ & \circ \end{array}\right]\left[\begin{array}{c} T_{1,j,k}^{n+1/3}\\ T_{2,j,k}^{n+1/3}\\ T_{3,j,k}^{n+1/3}\\ T_{4,j,k}^{n+1/3}\\ .\\ .\\ .\\ T_{n+1,j,k}^{n+1/3} \end{array}\right]=\left[\begin{array}{c} b_{1}\\ b_{2}\\ b_{3}\\ b_{4}\\ .\\ .\\ .\\ b_{n+1} \end{array}\right]. \end{array}$$

Different heat transfer correlations have been used to determine the heat transfer coefficient at the surface of fuel pin. Thom correlation (see (20)) is used for nucleate boiling and modified Bromley correlation (see (21)) is used for film boiling:

(20) $$\begin{array}{c} h_{o}={\left(\frac{e^{p/6.2}}{0.79}\right)}^{4}\Delta T_{\mathrm{sat}}^{3}, \end{array}$$

(21) $$\begin{array}{c} h_{o}=0.62{\left[\frac{k_{g}h_{fg}\rho_{g}g\left(\Delta \rho_{Lg}\right)}{\mu_{g}L\Delta T_{\mathrm{sat}}}\right]}^{0.25}, \end{array}$$

where

(22) $$\begin{array}{c} L=2\pi \left(\frac{g_{c}\sigma }{g\left(\Delta \rho_{Lg}\right)}\right). \end{array}$$

### 2.4. Steady State Computation for Uniform and Nonuniform Heat Generation

**Figure 4:**
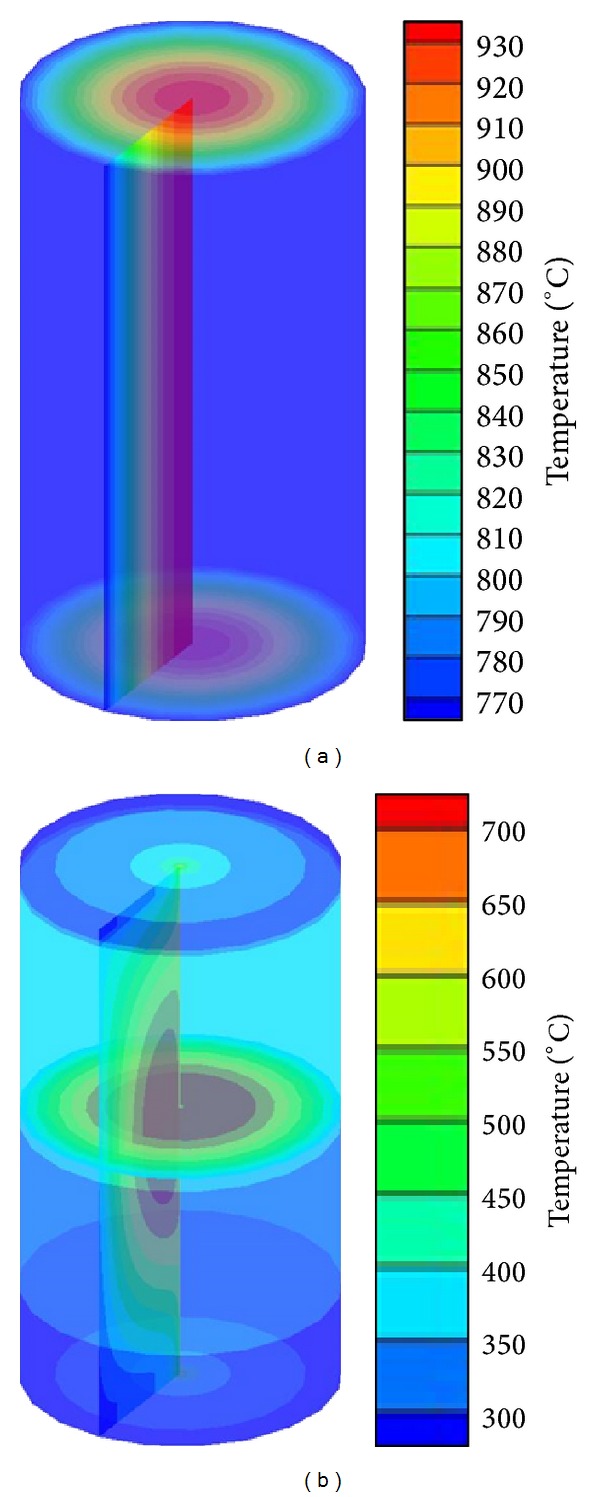
Temperature distribution on solid fuel pin in (a) uniform and (b) nonuniform (sinusoidal) heat generation.

Figure 4 shows the 3D temperature behavior in the fuel pin in different cases; the first case is uniform heat generation (1.16*E* + 8 W/m^3^) with a constant heat transfer coefficient and coolant temperature and second case is with sinusoidal heat generation with variation of coolant temperature along the length of the fuel pin.

Temperature in fuel pin has been predicted uniform across the fuel pin and at any cross section minimum and maximum temperatures are at the surface and centre of fuel pin, respectively. For sinusoidal heat generation, as shown in Figure 4, the fuel surface temperature is highest at the middle of the fuel pin since pin has a maximum heat flux. Temperature at any section of fuel pin along the length of fuel pin decreases in both directions from the middle of the fuel pin.

## 3. Benchmarking of 3D Model 

A benchmarking exercise has been carried out for the 3D conduction model with the available 1D analytical solution for a steady state case. As analytical solution of 3D is not available, the benchmarking is carried out with 1D solution. The analytical solution for estimating the coolant temperature and fuel pin surface temperature along the length of fuel pin is represented by (23) [19] and used to calculate the fluid temperature and surface temperature for 1D analytical solution. The heat flux along the fuel pin has been considered as a cosine profile. The Fourier conduction equation and its analytical for calculating the temperatures along the radius of fuel pin solution are given in (24) and (26), respectively.  Table 1 provides the input parameters for this exercise:

(23) $$\begin{array}{c} T_{\inf}=\frac{Q_{\max}AL}{\pi mc_{p}}\left(1-\cos\left(\frac{\pi x}{l}\right)\right)+T_{\text{in}},\\ T_{s}=\frac{Q_{\max}V}{h_{o}A}\sin\left(\frac{\pi x}{l}\right)+\frac{Q_{\max}V}{\pi mc_{p}}\left(1-\cos\frac{\pi x}{l}\right)+T_{\text{in}}. \end{array}$$

1D Fourier conduction equation is

(24) $$\begin{array}{c} \frac{1}{r}\frac{d}{dr}\left(r\frac{dT}{dr}\right)+\frac{Q^{\prime \prime \prime }}{K_{f}}=0. \end{array}$$

Apply boundary condition

(25) $$\begin{array}{c} {\frac{dT}{dr}|}_{r=0}=0. \end{array}$$

At outer surface.

Heat generation in body = heat transfer to coolant

(26) $$\begin{array}{c} Q^{\prime \prime \prime }\pi R_{f}^{2}l=2\pi R_{f}lh\left(T_{s}-T_{\inf}\right),\\ T\left(R\right)=\frac{Q^{\prime \prime \prime }R_{f}^{2}}{4K_{f}}\left(1-\frac{R^{2}}{R_{f}^{2}}\right)+T_{s}. \end{array}$$

**Figure 5:**
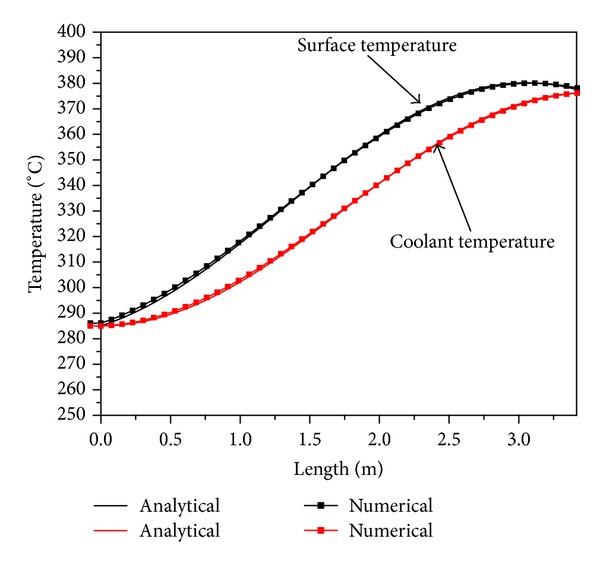
Benchmarking of coolant and surface temperature prediction.

Two-step benchmarking exercise has been done. In the first step, boundary conditions used for calculating the fuel radial temperature distribution for 3D model and analytical solution are compared and in the subsequent step the radial temperature distribution is compared.  Figure 5 shows the coolant temperature and fuel surface temperature variation along the length of the fuel pin.

**Figure 6:**
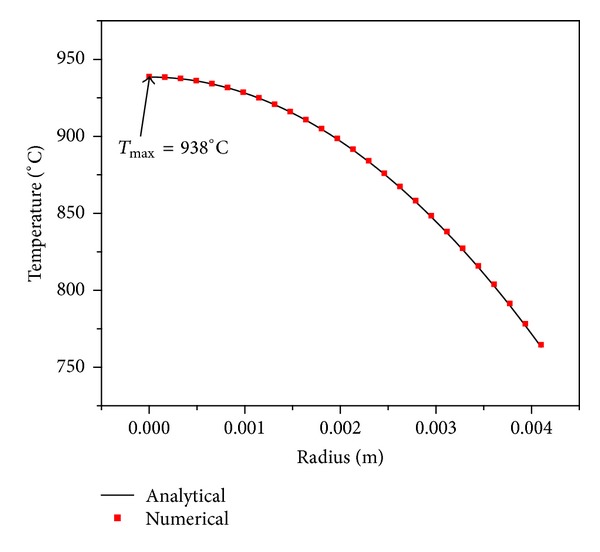
Steady state temperature distribution along the centre of fuel pin.

The calculated coolant temperature and surface temperature using (17) and (18) act as boundary conditions for the 3D code. A good agreement is found between the numerical code result with analytical solution obtained using (23).  Figure 6 shows the comparison of temperature distribution in *r*-direction with 3D numerical model and analytical solution for fuel rod. Result shows a good agreement between the two solutions.

**Figure 7:**
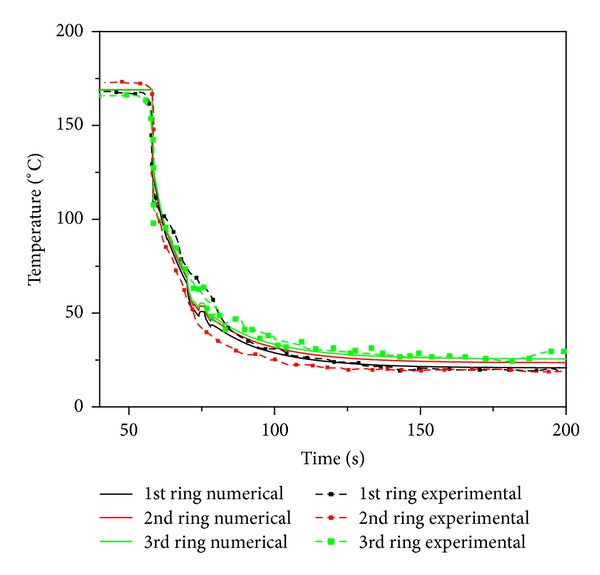
Benchmarking radial fuel surface temperature prediction of different rings.

Further, this single pin model has been extended to multiplepins model. The multiplepin model considers that pins are arranged in three concentric rings with radial jet simulation. A validation exercise has been carried out with experimental results reported by Patil N. D. The experimental case with initial temperature of 168°C for all pins with an injection flow rate of 73 lpm has been considered. The validation exercise results are shown in Figure 7 at an elevation of 2.4 m.

The exercise shows that the multipin model is able to capture the rewetting pattern well. However, in this study circumferential rewetting pattern could not be produced as measurements across the circumference of a simulated fuel pin are not being done/reported.

## 4. Study on Influence of Radial Rewetting on Conduction Heat Transfer Assessment 

**Figure 8:**
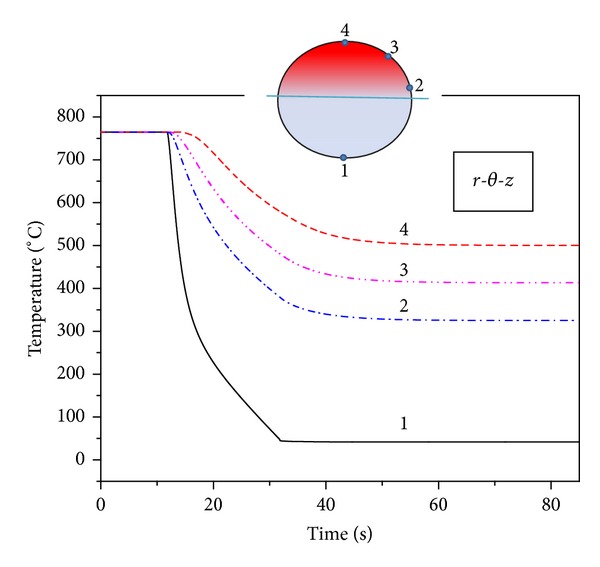
Transient surface temperature along the circumference of fuel pin.

As the circumferential conduction phenomenon is predicted by numerical technique, influence of ADI solution technique on the above mentioned findings is investigated. The schematic for radial reflooding is shown in Figure 12. For this study, boundary conditions like high rewetting heat transfer coefficient of 30000 W/m^2^ K (only water) are applied over half of the circumference (180°) of fuel pin where the water jet is likely to impinge. At the same time, a low heat transfer coefficient of 500 W/m^2^ K (only steam) is applied on the other half of the circumference (180°) where the jet is unlikely to reach. A linear variation of heat transfer coefficient from 500 W/m^2^ K (only steam) to 30000 W/m^2^ K (only water) for the water impinged surface is considered as described earlier. Variation of fluid temperature from 300°C (only steam) to 30°C (only water) is assumed over 20 s period at the node where rewetting front has reached.  Figure 8 shows the transient cooling temperature for four circumferential locations covering the half of the circumference which experienced the water jet.

**Figure 9:**
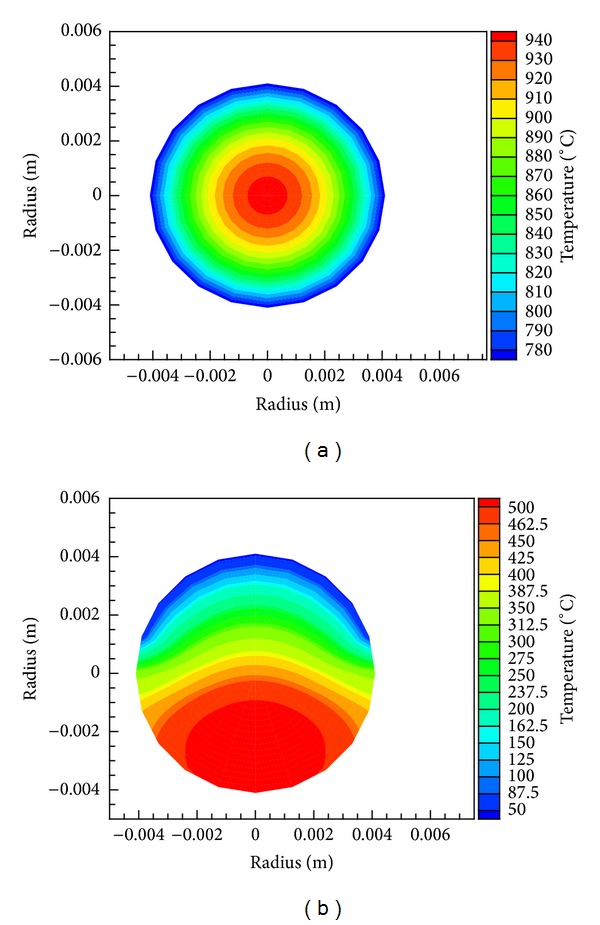
Influence of circumferential rewetting on fuel centre line temperature.

The result shows that for the chosen boundary conditions, influence of circumferential conduction is significant. Temperature of the unwetted portion dropped by 250°C with rewetting of the other half of the fuel over a minute of the beginning of rewetting of font section. The influence of one-sided rewetting has caused the maximum temperature of fuel pin to shift from centre to unrewetted portion of fuel pin, as shown in Figure 9.

## 5. Study on Influence of Cross Flow in Fuel Bundle

**Figure 10:**
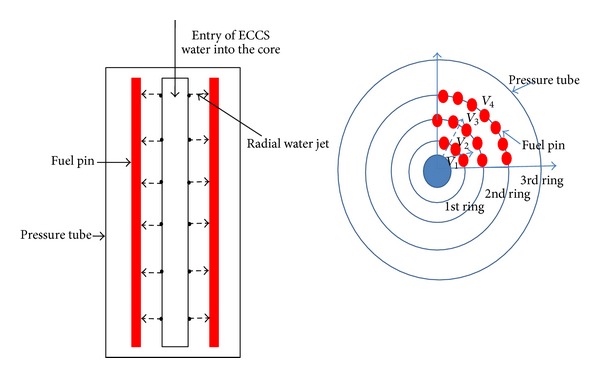
Influence of circumferential rewetting on fuel centre line temperature.

This work has been extended to multipins which are arranged in three concentric rings. The (1/4)th portion of fuel bundle, direction of radial injection of coolant flow, is shown in Figure 10.

To study the influence of the cross flow and axial flow on rewetting of fuel pin in coolant channel, the four and five control volumes have been modeled in radial and axial direction, respectively. The (1/5)th length of fuel pins is attached to one axial control volume. The innermost volume in radial direction is attached with only half of the total heat from 1st ring of fuel pin, whereas the second radial volume receives heat from remaining portion of the total heat from 1st ring of fuel and heat from front side of 2nd ring. It is a similar way of modeling for third and fourth volumes. In the axial direction, each innermost volume receives coolant from water tube. The coolant then goes into radial as well as axial direction. In top control volume coolant is heated by hot fuel pins and comes down due to gravitational force, mixes with cold coolant injected into second volume. In radial flow modeling, coolant first gets heated from first volume and then heated fluid enters into second volume and similar way to third and fourth volumes. In this flow distribution, top volume receives lowest coolant and next volume just below top volume gets higher, bottom most control volume receives highest amount of coolant. Two cases have been considered for modeling of power in the fuel pins; in the first case all pins in different rings have uniform power and in second case radial power variation (0.69 : 0.79 : 1.33) in different fuel rings has been considered.

**Figure 11:**
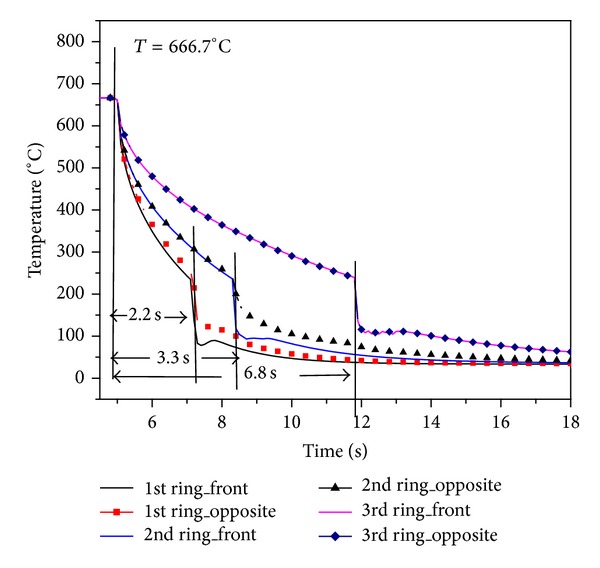
Influence of cross flow on rewetting of fuel surface temperature having uniform power.

**Figure 12:**
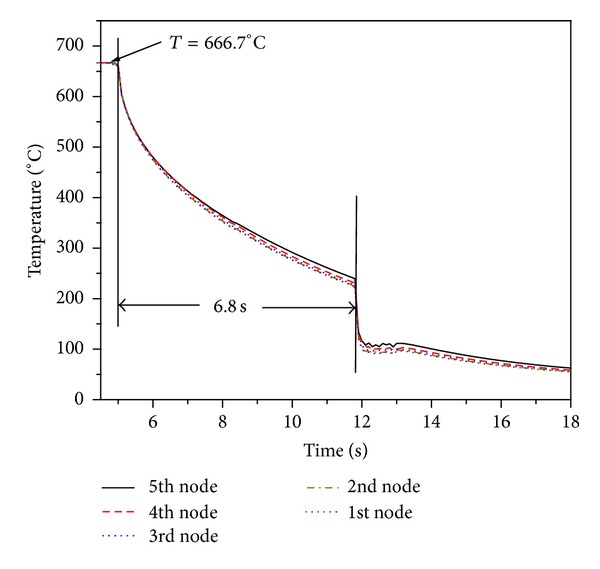
Transient temperature of different axial nodes of outer pin.

Figure 11 shows the fuel surface temperature behavior in different rings. Initial temperature of fuel pins in all the rings is 666.7°C. The coolant flow rate for rewetting is considered to be 90 lpm [20]. Fuel surface temperature starts decreasing with injection of coolant into the coolant channel. Inner ring pins quenching takes place at *t* = 2.2 s. on both sides of fuel pin. Due to the influence of circumferential conduction, fuel surface temperature in opposite sides of fuel pins of first ring falls slower than front side. Quenching time for second ring and third rings is 3.3 s and 6.8 s, respectively. Initially surface temperature of fuel pin falls slowly since heat transfer coefficient is poor due to the presence of strong steam film between heated surface and coolant. Once fuel surface temperature reaches below the Leidenfrost point, surface gets rewetted which results in rapid fall of fuel surface temperature. The axial fuel surface temperature of outer ring at different locations is shown in Figure 12. Rewetting time of all nodes is close to *t* = 6.8 s but temperature fall rate at top node is a little slower than lower node. It is due to the lower coolant amount received by top node.

**Figure 13:**
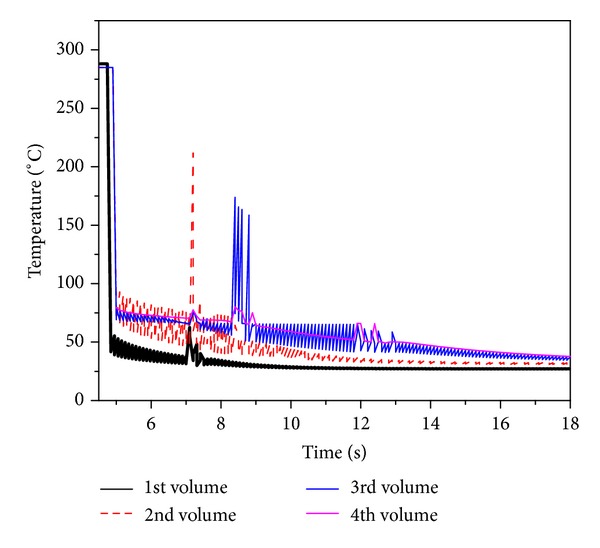
Transient coolant temperature in different volume.

During quenching of inner ring fuel pins, the heat released from the fuel pins is more as compared to the outer ring fuel pins. Therefore, the rise in coolant temperature observed during the quenching period is more for the inner fuel pins.  Figure 13 shows the coolant temperature in different volume. The average temperature of coolant rises when it moves from first volume to second volume and continues till the last volume in radial direction. Fourth volume gets maximum coolant temperature. Oscillation of coolant temperature is due to continuous supply of coolant to volume.

**Figure 14:**
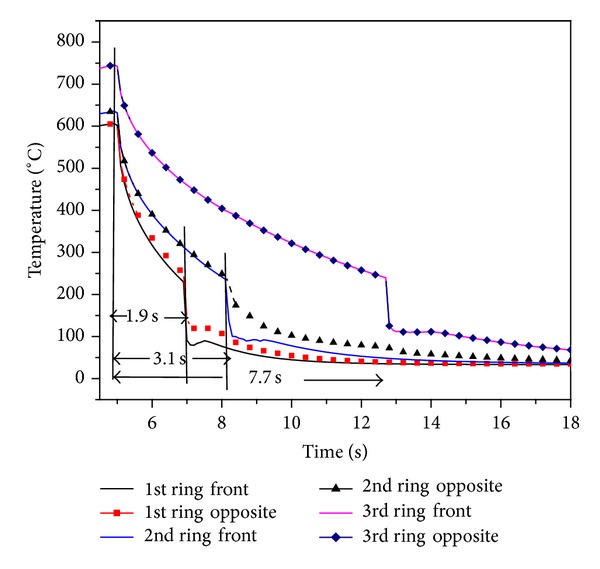
Influence of cross flow on rewetting of fuel surface temperature having radial power.

For the case of rewetting with radial power distribution, the initial temperatures obtained in 1st, 2nd, and 3rd rings are 605°C, 635°C, and 742°C [16], respectively, as shown in Figure 14. The same mass flow rate (90 lpm) has been considered for quenching of fuel pins. Similar to the previous case, during initial period of quenching, strong steam film is formed between the fuel surface and liquid so that the fuel surface temperature decreases slowly because of poor heat transfer. Once surface temperature reaches Leidenfrost point it rewets the surface. The rewetting time of heated fuel surface temperatures is 1.9 s, 3.1 s, and 7.7 s. Influence of circumferential conduction effect has been also observed. Front side clad surface temperature falls faster than opposite side.

## 6. Conclusion 

Numerical studies on the influence of circumferential effect and cross flow for AHWR fuel bundle reflooding phenomena has been studied with the help of 3D conduction numerical model. A circumferential conduction is found to be significant for radial jet rewetting. The conservative assessment shows that radial jet is able to bring down the unrewetted portion temperature by 250°C. A radial direction jet is found to be effective to cool the hot surface. The numerical studies on influence of cross flow have been studied. It has been found that even for the clad surfaces having the same initial surface temperature, rewetting time of inner fuel pins is lesser than the outer pins and influence of circumferential conduction has been also observed. The axial fuel surface temperature behavior at different locations is observed to be nearly same.

## Figures and Tables

**Table 1:** Input data for benchmark exercise.

Parameter	Value
Radius of fuel pin	0.0041 m
Length of fuel pin	3.5 m
Fuel thermal conductivity	2.8 W/m K
Fuel density	10730 kg/m^3^
Fuel specific heat	0.28 KJ/kg K
Inlet coolant temperature	300°C
Heat transfer coefficient at surface	500 W/m^2^K
Axial heat generation rate	3.2 × 10^8^sin⁡(π*x*/*l*) (W/m^3^)

## Nomenclature

### Latin Symbols

*A*: Area (m^2^)
*C*_*p*_: Specific heat capacity (J/kgK)
*g*: Local acceleration due to gravity (m/s^2^)
*g*_*c*_: Gravitation constant (m/s^2^)
*h*_*o*_: Heat transfer coefficient at outer surface (W/m^2^K)
*h*_*fg*_: Latent heat of vaporization (W/m)
*k*_*g*_: Thermal conductivity of steam (W/mK)
*k*_*f*_: Thermal conductivity of fuel (W/mK)
*l*: Length of fuel (m)
*m*: Coolant flow rate (kg/s)
*P*: Pressure (MPa)
*Q*′′′: Local heat generation in fuel (W/m^3^)
*r*: Fuel radius changing in *r*-direction
*R*_*f*_: Outer radius of fuel pin (m)
*T*: Temperature (K)
*T*_*s*_: Fuel surface temperature (K)
*T*_out_: Coolant outlet temperature (K)
*T*_in_: Coolant inlet temperature (K)
*T*_inf⁡_: Average coolant temperature
Δ*T*_sat⁡_: Temperature difference between fuel surface and saturation temperature of coolant (°C)
*t*: Time (s)
Δ*t*: Time increment (s)
*V*: Volume (m^3^)
Δ*r*: Distance increment in fuel (m)
Δ*z*: Distance increment in *z*-direction (m).

### Greek Symbols

*ρ*_*g*_: Density of steam (kg/m^3^)
*ρ*: Density fuel (kg/m^3^)
*σ*: Surface tension (N/m)
*θ*: Angle (radians)
*α*: Thermal diffusivity (m^2^/s)
Δ*ρ*_*Lg*_: Density difference between liquid and steam (kg/m^3^)
Δ*θ*: Angle increment in circumference direction (radians).

### Other Symbols

$F_{0}=\left(\frac{k_{f}\Delta t}{\rho C_{p}{\left(\Delta r\right)}^{2}}\right)$
$F_{\theta }=\left(\frac{k_{f}\Delta t}{\rho C_{p}{\left(\Delta \theta \right)}^{2}}\right)$
$F_{z}=\left(\frac{k_{f}\Delta t}{\rho C_{p}{\left(\Delta z\right)}^{2}}\right)$: .

### Subscripts

*f*: Fuel
*i*: Mesh point in *x*-direction
*j*: Mesh point in *θ*-direction
*k*: Mesh point in *z*-direction.

### Superscript

*n*: Time step.
